# Establishment of quantitative indicators for an efficient treatment on masticatory muscle pain

**DOI:** 10.1002/cre2.705

**Published:** 2022-12-23

**Authors:** Sunhee Lee, Hye‐Min Ju, Donald Ho, Byong‐Sop Song, Sung‐Hee Jeong, Yong‐Woo Ahn, Soo‐Min Ok, Kyounga Cheon

**Affiliations:** ^1^ Department of Oral Medicine, Dental and Life Science Institute Pusan National University Yangsan Republic of Korea; ^2^ Dental Research Institute, Pusan National University Dental Hospital Yangsan Republic of Korea; ^3^ Department of Pediatric Dentistry University of Alabama at Birmingham Birmingham Alabama USA; ^4^ Department of Statistics Graduate School of the Pusan National University Busan Republic of Korea

**Keywords:** differential diagnosis, individualized treatment, masticatory muscle pain, temporomandibular joint disorder

## Abstract

**Background:**

Although several studies have investigated effective treatments for masticatory muscle pain (MMP), no unified conclusion has been drawn regarding the effectiveness of these treatments.

**Objectives:**

This study aimed to define quantitative indicators for predicting the outcome of MMP treatment.

**Materials and Methods:**

In total, patients aged 20–70 years were recruited and divided into the MMP (*n* = 24) and control (*n* = 36) groups, based on the presence of MMP according to the Diagnostic Criteria for Temporomandibular Disorders. At pretreatment, the MMP group was assessed using quantitative indicators such as subjective pain levels, pain duration, graded chronic pain scale (GCPS), and perceived stress scale (PSS). Salivary alpha‐amylase (sAA) and interleukin‐6 (IL‐6) levels were analyzed. The masticatory muscle palpation score and the range of mouth opening were measured. At posttreatment, subjective pain levels, mouth opening, and treatment/medication duration were examined. The PSS and sAA levels were assessed in the control group.

**Results:**

sAA levels in the MMP group were significantly higher than those in the control group (*p* < .05). The masseter muscle palpation score (MPS) showed a positive correlation with IL‐6 levels (ρ = 0.503, *p* < .05) and a negative correlation with nonsteroidal anti‐inflammatory drug (NSAID) treatment period (ρ = −0.462, *p* < .05). The temporalis muscle palpation score (TPS) was positively correlated with pain duration and GCPS grade (ρ = 0.483, *p* < .05, and ρ = 0.445, *p* < .05, respectively).

**Conclusions:**

Treatment with NSAIDs was effective in the MMP group with high MPS and IL‐6 levels, but not in the MMP group with high TPS, pain duration, and GCPS grade.

## 1 INTRODUCTION

Masticatory muscle disorder is a subgroup of temporomandibular disorders (TMDs), with a prevalence of 31.4%–88.7% among TMDs (Reiter et al., 2012). The causes of masticatory muscle pain (MMP) are diverse, with combinations of physical, behavioral, social, and psychological factors (Leeuw & Klasser, 2018). Although several studies have investigated effective treatments for MMP, no unified conclusion has been drawn regarding the effectiveness of these treatments (Calixtre et al., 2015; List & Axelsson, 2010), primarily due to the difficulty in accurately classifying, diagnosing, and treating patients.

According to the Diagnostic Criteria for TMD (DC/TMD), practitioners are advised to conduct multidimensional interviews and evaluate patients, to determine specific pain patterns (i.e., acuteness or chronicity; Assadourian et al., 2020; Schiffman et al., 2014). However, most interviews often depend on patients' memory and cognitive characteristics, limiting the prospects of obtaining reliable and accurate information. Discrepancies between self‐perceived symptoms and biological indicators may reflect symptoms (Assadourian et al., 2020). Subjective symptoms should be considered when diagnosing MMP; however, quantitative indicators that objectify the differences in individual treatment responses should be used as well. Nevertheless, current diagnostic methods are not clearly developed using quantitative indicators (Masuda et al., 2018).

Acute MMP symptoms can be easily treated with medication and physical treatment. However, chronic MMP symptoms are more complicated, require various examinations, and are not easily treated (Okeson, 2019). If it is not accurately diagnosed, the symptoms can last longer and progress to involve the central nervous system, and recovery becomes difficult with longer treatment periods and poor prognosis. One third of patients with chronic MMP experience pain lasting 8–10 years (Von Korff et al., 1993). Additionally, a vicious cycle of worsening mental stress may lead to depression and anxiety (Okeson, 2019).

Centralization of pain results from changes in the properties of neurons in the central nervous system caused by inflammation, neural injury, and prolonged pain. Centralization of pain through neuropathic changes may be present even with a short pain history, and not available to fully diagnose with current diagnostic methods (Institute of Medicine Committee on Advancing Pain Research Care Education, 2011; Verhaak et al., 1998). Three months is not an absolute criterion for dividing acuteness and chronicity. By defining indicators that can reflect disease characteristics, the appropriate treatment method can be selected (Treede et al., 2015). Diagnosing accurate chronicity and predicting prognosis improve patient coordination and trustworthiness to healthcare professionals. Thus, the development of various quantitative indicators measurable at pretreatment could assist to predict prognosis accurately and determine personalized treatment.

Salivary cytokines can be noninvasive indicators to diagnose acute inflammation (Slavish et al., 2015). Among them, interleukin‐6 (IL‐6), a proinflammatory cytokine, is an important mediator of the acute phase inflammatory response (Hunter & Jones, 2015). It is also known as a myokine secreted directly from muscles (Lightfoot & Cooper, 2016). Pain caused by acute inflammation is alleviated by nonsteroidal anti‐inflammatory drugs (NSAIDs; Berg et al., 1999). However, when pain is chronically protracted, spontaneous pain is caused by centralization, regardless of peripheral inflammation, and not alleviated by NSAIDs (Knights et al., 2010). Therefore chronicity of pain should be treated with a multifaceted approach such as consultation, medication, and physical therapy (Laskin et al., 2006). The classical method to evaluate chronicity is an interview regarding pain duration, with simple questions such as “How many years or months ago did the pain begin?” (Schiffman et al., 2014) However, chronicity cannot simply be diagnosed based on pain duration alone. Various factors, such as pain characteristics, quality of life, and psychosocial factors, should be considered (Voscopoulos & Lema, 2010). Among the survey questionnaires, the graded chronic pain scale (GCPS) is a reliable, systematic, and widely used questionnaire for chronic pain (Von Korff et al., 1992). Despite current limitations on subjective self‐report, the GCPS could provide characteristics of pain (Hawker et al., 2011). Furthermore, it is difficult to distinguish between borderline symptoms of acuteness and chronicity. Therefore, GCPS is implemented to define quantitative indicators to diagnose characteristics of pain and differentiate treatment modalities (i.e., NSAIDS, and multifaced treatment modalities such as naturopathy, neurology, physical and massage therapy, and psychology) in acute and chronic MMP.

Chronic pain and chronic stress may result in overlapping physiological pathways (Abdallah & Geha, 2017). Furthermore, stress is known to cause musculoskeletal pain (Lang et al., 2012) because sympathetic nerve activity is elevated by mental stress (Nater et al., 2006), which directly affects human muscles (Radovanovic et al., 2015). A recent study reported that 49.8% of orofacial pain patients experienced traumatic life events (de Leeuw et al., 2005), and 15%–23% of patients with orofacial pain showed clinically significant symptoms of post‐traumatic stress disorder (Burris et al., 2009). Questionnaires from the patients' surveys, such as the perceived stress scale (PSS), can be used to determine the degree of stress in patients (Park & Colvin, 2019). However, objective stress indicators may be required because some patients may not realize the actual extent of their stress. In addition, because the illiteracy rate of the world's population aged 15 years or above is 13.7%, according to the latest data from the UNESCO Institute for Statistics, the development of objective stress indicators that do not depend on written questionnaires is essential (UIS, 2019). Meanwhile, salivary alpha‐amylase (sAA) is widely used as a noninvasive and convenient objective measure of sympathetic hyperactivity in chronic diseases (e.g., mental illness; Nater & Rohleder, 2009). Chronic stress increases the production and storage of catecholamines (McCarty et al., 1988). Thus, sAA activity could be an effective indicator for measuring stress from a more objective perspective.

Among the masticatory muscles, the masseter and temporalis muscles can be examined by palpation. They are both jaw‐elevating muscles, however, those masticatory muscles differ in the muscle fibers distribution and fatigue resistance (Isola et al., 2018) which affect the symptoms and prognosis of MMP (Scott et al., 2001). Depending on the type of muscle affected by pain, personalized treatment should be applied. Therefore, this study hypothesized that the degree of chronicity is dependent on the painful muscle region.

This study aimed to define quantitative indicators that reflect the characteristics of MMP and to develop ground data for predicting treatment outcomes of MMP, even in short‐duration cases.

## 2 MATERIALS AND METHODS

### 2.1 Recruitment of research subjects

The recruitment notice was posted to recruit volunteers from August 21, 2017, to March 8, 2018. Among the patients aged 20–70 years who visited the Department of Oral Medicine at Pusan National University Dental Hospital, subjects with MMP (*N* = 68) were included in the MMP group, and those without MMP (*N* = 65) were included in the control group based on DC/TMD (Schiffman et al., 2014). Fifty‐eight subjects in the MMP and control groups were excluded based on the following exclusion criteria: (1) any medical problem; (2) active inflammatory conditions, including periodontitis; (3) oral disease; (4) oral and maxillofacial trauma; (5) food intake, exercise, smoking, drinking alcohol, or caffeine intake within 1 h; (6) lack of sleep the previous night; and (7) refusal to participate in the study after providing consent (Nater et al., 2007). Subsequently, 15 subjects in the MMP group were lost to follow‐up due to personal circumstances, such as moving or busy schedules. Finally, we analyzed 24 MMP participants and 36 controls.

**Table 1:** Quantitative indicators from measurements of MMP group and control group

	MMP group	Control group
**Pretreatment**		
Patients' survey	Gender	Gender
	Age	Age
	Subjective pain level (numerical rating scale [NRS])	
	Score of pain duration (Dur)	
	Graded chronic pain scale (GCPS)	
	Perceived stress scale (PSS)	Perceived stress scale (PSS)
Collected saliva analysis	Salivary alpha‐amylase (sAA)	Salivary alpha‐amylase (sAA)
	Interleukin‐6 (IL‐6)	
Clinical examination by a skilled clinician	Masticatory muscle palpation score (Masseter/Temporalis)	
	Initial pain‐free opening (PFO‐initial) Initial maximum assisted opening (MAO‐initial)	
**Posttreatment**		
Patients' survey	Subjective pain level (numerical rating scale [NRS])	
Clinical examination by a skilled clinician	Final pain‐free opening (PFO‐final) Final maximum assisted opening (MAO‐final)	
Treatment review	Treatment/medication duration	

### 2.2 Diagnostic measurements (Table 1)

#### 2.2.1 MMP groups at pretreatment

Sex and age were recorded through a basic survey. The duration of pain from onset to the first visit (hereinafter referred to as “Dur” in this study) was recorded. In the survey, Dur was numerically rated from 1 to 4 (1: within 1 month; 2: 1−6 months; 3: 6−12 months; and 4: over 1 year; Table 1).

The GCPS and PSS were assessed. GCPS was used as the main measure of pain intensity and disability due to pain. The GCPS comprises three questions related to pain intensity and four questions related to disability due to pain. Subsequently, we calculated the pain intensity score using questions 1−3, the disability score using questions 4−6, and the disability days using question 7. Disability points were calculated by combining disability scores and disability days. The pain intensity score and disability points determined GCPS grades from 1 to 4 (Von Korff et al., 1992). We used the pain intensity, disability score, disability days, disability points, and GCPS grades as variables of chronicity. For stress measurements, a PSS survey was used.

Saliva was collected from 13:30 to 16:30, a relatively stable period for sAA activity, (Nater et al., 2007) and IL‐6 concentration throughout the day (Nilsonne et al., 2016). The oral cavity was rinsed with water to flush out the food components before saliva collection. For each participant, 3 ml of unstimulated whole saliva was collected by dropping it into a 15‐ml tube and immediately stored at −70°C before the assay. After thorough thawing, the samples were vortexed and centrifuged, and sAA activity was assayed using a commercial kinetic enzyme assay kit (Salimetrics®; LLC, USA), and IL‐6 concentrations were determined using a commercial ELISA kit (Salimetrics®; LLC, USA; Nater et al., 2007; Nilsonne et al., 2016; Rohleder & Nater, 2009).

The regional palpation scores of the masseter and temporalis muscles were recorded using quantitative evaluation. First, the left and right sides were palpated simultaneously for 5 s using 1 kg/cm^2^ pressure for each muscle separately (method for evaluating chronic myalgia based on DC/TMD; Schiffman et al., 2014). Next, the patient's reaction was numerically rated from 0 to 3 points (0: no pain or tenderness; 1: tenderness; 2: definite discomfort or pain; and 3: evasive action or tearing or desire not to be palpated again; Okeson, 2019). Finally, the presence of any referred pain was recorded by asking, “Did you feel pain just under my finger, or did you also feel it somewhere else?” (Schiffman et al., 2014).

The main symptoms of MMP are opening limitation and pain. Therefore, improvements in MMP can be evaluated by comparing the range of mouth opening or pain level at the first visit and at the end of treatment. The current subjective pain levels were scored from 0 to 10 on a numerical rating scale (NRS). In this study, the term “NRS‐initial” was used as the initial NRS. The range of mouth opening was measured as the initial pain‐free opening (PFO‐initial) and initial maximum assisted opening (MAO‐initial) based on DC/TMD (Schiffman et al., 2014).

#### 2.2.2 MMP group at the post‐treatment

Final NRS (NRS‐final), final pain‐free opening (PFO‐final), and a final maximum assisted opening (MAO‐final) were measured. Furthermore, the total duration of treatment was recorded as a measure of how difficult it was to recover from pain (treatment period, Tx period). The dosing period of the NSAIDs (Brufen®; ibuprofen, oral 400 mg, three times a day, Samil Co.) required to evaluate the amount of NSAIDs to resolve the MMP was also recorded (NSAID‐period). Only prescribed routine medication (ibuprofen) was allowed, and no other drugs were administered to any patient. All patients were educated on TMD self‐care instructions and treated with physical therapy, including low‐level laser therapy (880 nm, 5 min), ultrasound therapy (3 MHz, 1 W/cm^2^, 5 min), and transcutaneous electrical nerve stimulation (10–30 Hz, 20 min) at every visit.

#### 2.2.3 Control group

As in the MMP group, gender and age were recorded through a basic survey of the controls. For stress measurements, a PSS survey was used. Saliva samples were used to analyze sAA in the same way as in the MMP group (Nater et al., 2007; Rohleder & Nater, 2009).

### 2.3 Statistical analysis

The Mann–Whitney *U* test and the chi‐square test were performed to examine the differences in age and sex ratio between the MMP and control groups. The Mann–Whitney *U* test was performed to examine the differences in sAA and PSS between the MMP and control groups. Linear regression analysis was used to evaluate the association of disability scores with sAA and IL‐6 levels. A scatter plot with two axes of sAA and IL‐6 was plotted to examine their relationship. After dividing the data into quadrants, the Mann–Whitney *U* test was performed to assess whether there were differences in the chronicity variables among the quadrant groups. Mann–Whitney *U* test and Spearman's rho correlation analysis were performed to analyze the correlation between the regions of the muscle and quantitative indicators. It was also used to analyze the correlation between PSS and chronicity. To determine the indicators to be used for predicting prognosis, Spearman's rho correlation analysis was performed for recordable variables at the initial visit and indicators associated with treatment outcomes (PFO‐final and NRS‐final, respectively). Prognosis predictive formulas were created using multiple linear regression analysis using indicators that were relevant to the correlation analysis. All data analyses were performed using IBM® SPSS® Statistics software version 21 (IBM Corp., Armonk, NY, USA). Statistical significance was set at *p* < .05.

## 3 RESULTS

### 3.1 Demographics

Table 2 presents the demographic characteristics of the participants. There was no significant difference in age or sex distribution between the MMP and control groups.

**Table 2:** Participants' characteristics

	MMP group	Control group	*p* Value
Age, mean ± SD (years)	41 ± 19	32 ± 12	.482^a^
Gender ratio (F:M)	17:7	22:14	.439^b^
Sample size	24	36	–

Abbreviations: p, significance; SD, standard deviation.

^a^ Determined from the Mann–Whitney *U* test.

^b^ Determined from the *χ* ^2^ test.

Table 3 shows the baselines and changes in pre‐ and posttreatment data in the MMP group. The NRS of posttreatments significantly improved compared to the NRS of pretreatments, and no significant differences were found between PFO and MAO pre‐and post‐treatment. The Dur in the MMP group was 2.5 ± 1.38; therefore, most cases can be regarded to have pain for a long duration of more than 6 months.

**Table 3:** MMP group' baselines and changes of pre‐ and posttreatments

(Mean ± SD)	Pretreatment	Posttreatment	*p* Value
NRS	4.92 ± 1.96	3.30 ± 2.48	.032
PFO (mm)	36.44 ± 11.36	38.67 ± 8.58	.256
MAO (mm)	42.69 ± 8.53	43.92 ± 7.11	.317
Dur	2.5 ± 1.38		–
Tx‐period (month)	8.39 ± 7.61		
NSAID‐period (week)	5.37 ± 4.96		
% of improved	54.17 (13/24)		

Abbreviations: Dur, from onset to the first visit, numerically rated from 1 to 4 (1, within 1 month; 2, 1–6 months; 3, 6–12 months; and 4, over 1 year); MAO, maximum assisted opening; NRS, numerically rated scale of pain; NSAID‐period, dosing period of NSAIDs; p, significance (Mann–Whitney *U* test); PFO, pain‐free opening; SD, standard deviation; Tx period, treatment period; % of improved, Percentage of improvement based on PFO in the MMP group.

### 3.2 SAA and IL‐6 activity associated with GCPS disability score

**Figure 1:**
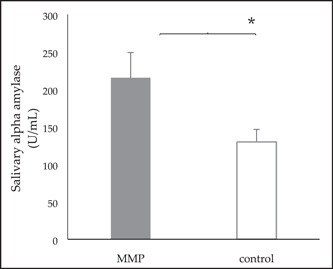
Salivary alpha‐amylase activity in the MMP group is significantly higher than that in the control group (*p < .05, Mann–Whitney *U* test). Error bars indicate the standard error of the mean.

**Figure 2:**
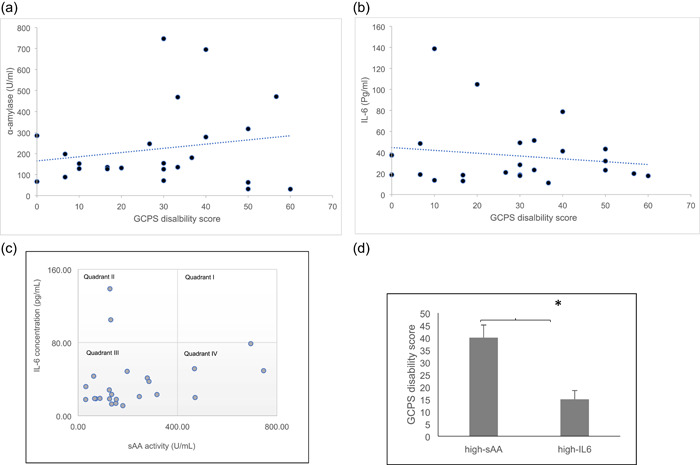
Linear regression analysis was used to evaluate the association of disability score with α‐amylase, *p* > .05 (a) and IL‐6, *p* > .05 (b) (*n* = 24). (c) Scatter plot with two axes, salivary alpha‐amylase (sAA) activity and interleukin‐6 (IL‐6) concentration in saliva, in the MMP group (*N* = 24). Data were divided into four quadrants, with IL‐6 ranging from 0 to 80 pg/ml and 80 to 160 pg/ml and sAA ranging from 0 to 400 U/ml and 400 to 800 U/ml. In most cases, both IL‐6 and sAA are low (quadrant III). In some cases, only IL‐6 (quadrant II) or sAA is hyperactive (quadrant IV). None of the patients show simultaneously hyperactive IL‐6 and sAA (quadrant I). (d) Comparing the MMP group's quantitative indicators between quadrant II (high‐IL‐6) and quadrant IV (high‐sAA), the disability scores in the GCPS category are found to be significantly higher in the quadrant‐IV (high‐sAA) group (*p < .05, Mann–Whitney *U* test). Error bars indicate the standard error of the mean.

The sAA activity was significantly higher in the MMP group than in the control group (*p* < .05) at pretreatment, which suggests the occurrence of sympathetic hyperactivity in the MMP group (Figure 1). Although sAA was not significantly correlated with other indicators in the MMP group (*p* > .05), it showed a weak positive correlation with the GCPS disability score, which was a chronic factor (*p* > .05; Figure 2a). In contrast, IL‐6 showed a very weak negative correlation with disability score (*p* > .05; Figure 2b).

A scatter plot (Robert et al., 2017) of the relationship between sAA and IL‐6 levels was created. When the data were divided into quadrants, a specific pattern was observed. There were many MMP groups marked in quadrant III with low IL‐6 and sAA levels. Several MMP groups were marked in quadrant II with low IL‐6 and hyperactive sAA and in quadrant IV with low sAA and hyperactive IL‐6. However, there were no MMP groups in quadrant I, where both IL‐6 and sAA were hyperactive (Figure 2c).

The quantitative indicators of the MMP group in quadrants II and IV were examined for significant differences between the two groups. The GCPS disability score was significantly higher in quadrant IV (high‐sAA) patients than in quadrant II (high‐IL‐6) patients (*p* < .05; Figure 2d).

PSS scores were not correlated with sAA, IL‐6, or any other quantitative indicators (data not shown).

### 3.3 Muscle indicators to determine acuteness or chronicity

The correlations between quantitative indicators and the palpation score for each masticatory muscle, temporalis, and masseter muscle were analyzed (Table 4). Masseter muscle palpation score (MPS) was positively correlated with IL‐6 (acute inflammatory factors; *p* < .05) and negatively correlated with the dosing period of NSAIDs (*p* < .05). Quantitative indicators related to chronicity were not related to the MPS levels. The temporalis muscle palpation score (TPS) positively correlated with chronicity factors, such as pain duration (Dur) and GCPS grade. IL‐6 and the dosing period of NSAIDs were not related to TPS.

**Table 4:** Spearman's correlation between the palpation score of each muscle and variables of chronicity and inflammation

Palpation score		Chronicity variables			Inflammatory variables	
		Dur	GCPS	Disability score	IL‐6	NSAID‐period
MPS (*n* = 12)	ρ	.280	−.099	−.045	.503	−.462
	*p*	.185	.645	.993	.012*	.026*
TPS (*n* = 8)	ρ	.483	.445	.223	−.178	−.115
	*p*	.017*	.029*	.019*	.404	.600

Abbreviations: Dur, pain duration; GCPS, graded chronic pain scale; IL‐6, interleukin‐6 concentration; MPS, masseter muscle palpation score; NSAID‐period, dosing period of NSAIDs.; *p*, significance (two‐tailed); ρ, Spearman's correlation coefficient; TPS, temporalis muscle palpation score.

* *p* < .05.

**Figure 3:**
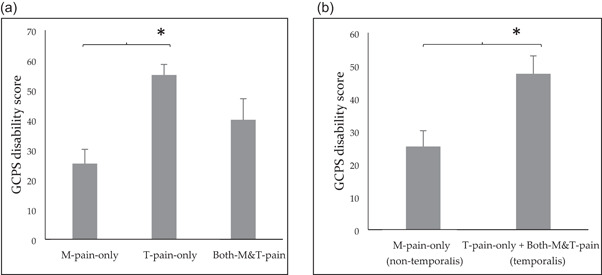
(a) The graded chronic pain scale (GCPS) disability score is significantly different between the M‐pain‐only (only masseter pain group) and T‐pain‐only (only temporalis pain group) groups (*p < .05, Mann–Whitney *U* test), while there is no difference between the both‐M&T‐pain (both masseter and temporalis pain group) and T‐pain‐only groups or both‐M&T‐pain and M‐pain‐only groups (*p* > .05). (b) There is a significant difference in the GCPS disability score between the M‐pain‐only group (painless temporalis muscle group) and the combined T‐pain‐only and both M&T pain groups (painful temporalis muscle group; *p* < .05). Error bars indicate the standard error of the mean.

The MMP group was divided into three subgroups to compare quantitative indicator characteristics according to the muscle regions responding to palpation. The group with pain only in the masseter muscle was called the M‐pain‐only group; the group with pain only in the temporalis muscle was called the T‐pain‐only group, and the group with pain in both regions was called the both‐M&T‐pain group. Pain was determined to be present if the palpation score was 1–3, and it was determined to be absent if the palpation score was 0. There was a significant difference in the GCPS disability score between the M‐pain‐only and T‐pain‐only groups (*p* < .05), but not between the both‐M&T‐pain and T‐pain‐only groups or both‐M&T‐pain and M‐pain‐only groups (*p* > .05; Figure 3a). The T‐pain‐only and both M&T pain groups were combined to create a painful temporalis muscle group, which showed a higher GCPS disability score than the painless temporalis muscle group, that is, the M‐pain‐only group (*p* < .05; Figure 3b). When the M‐pain‐only and both M&T pain groups were combined to create the painful masseter muscle group and compared to the painless masseter muscle group, that is, the T‐pain‐only group, there was no significant difference in the GCPS disability score between the two groups (*p* > .05; data not shown).

### 3.4 Prognosis prediction

#### 3.4.1 Correlation analysis of prognosis indicators with data detectable at the first visit

In Table 5, we analyzed whether PFO‐final and NRS‐final correlated with the data measured at pretreatment. The PFO‐final score had a significant positive correlation with the MPS. In the regression analysis, there was a significant negative correlation between PFO‐final and TPS. The analysis of the relationship of PFO‐final with the other indicators at posttreatment showed significant negative correlations with the Tx‐period and NSAID‐period, but no significant correlation with NRS‐final. NRS‐final had a significant correlation with NRS‐initial but no correlation with any other variable.

**Table 5:** Spearman's correlation of prognosis indicators with quantitative indicators at the first visit and at the end of treatment

Prognosis variables		Data at the first visit								Data at the end of treatment			
		MPS	TPS	GCPS	PFO‐initial	MAO‐initial	sAA	IL‐6	NRS‐initial	Tx‐period	NSAID‐period	NRS‐final	PFO‐final
PFO‐final	ρ	.474*	−.188	−.483*	.827**	.851**	.242	.061	.146	−.562*	−.578*	−.108	–
	*P*	.047	.456	.042	.000	.000	.333	.810	.563	.015	.012	.680	–
NRS‐final	ρ	.061	−.007	.161	−.245	−.049	.054	.370	.601*	.283	−.130	–	−.108
	*P*	.816	.977	.537	.343	.852	.836	.144	.011	.272	.620	–	.680

Abbreviations: GCPS, graded chronic pain scale; IL‐6, interleukin‐6 concentration; MAO‐initial, initial maximum assisted opening; MPS, masseter muscle palpation score; NRS‐initial, initial numerically rated scale of pain; NRS‐final, numerically rated scale of pain at the end of treatment; NSAID‐period, NSAID dosing period; p, significance (two‐tailed); PFO‐initial, initial pain‐free opening; PFO‐final, pain‐free opening at the end of treatment; ρ, Spearman's correlation coefficient (*p < .05; **p < .01); TPS, temporalis muscle palpation score; sAA, salivary alpha‐amylase activity; Tx‐period, treatment period.

#### 3.4.2 Regression analysis to establish the prognosis (PFO‐final) prediction formulas

Two multiple linear regression equations with (1) or without (2) GCPS, sAA, and IL‐6 were formulated to predict PFO‐final based on the detectable variables at the first visit (Formula 1: *R* ^2^ = .929, *F* = 18.643 [*p* = .000], Formula 2; Table 6; *R* ^2^ = .906, *F* = 31.267 [*p* = .000]). The addition of sAA, IL6, and GCPS did not cause many R2 changes in the model. The prognosis prediction Formulas (1) and (2) for PFO‐final are as follows:

(1) $$\text{PFO}-\text{final}=1.665+1.531*\text{MPS}-2.339*\text{TPS}-2.872*\text{GCPS}-0.002*\text{sAA}+0.008*\text{IL}6-0.666*{\text{NRS}}_{\text{initial}}+0.967*{\text{MAO}}_{\text{initial}}$$

(2) $$\text{PFO}-\text{final}=-0.589+2.049*\text{MPS}-3.038*\text{TPS}-0.975*{\text{NRS}}_{\text{initial}}+0.951*{\text{MAO}}_{\text{initial}}$$

**Table 6:** Factors associated with pain‐free opening in a multiple linear model, Formula (2)

	Unstandardized coefficients		Standardized coefficients	*t*	*p*	Collinearity statistics	
	*B*	SE	*β*			Tolerance	VIF
(Constant)	−0.589	4.333		−0.136	.894		
MPS	2.049	0.524	0.352	3.908	.002*	0.893	1.120
TPS	−3.038	0.712	−0.375	−4.268	.001*	0.938	1.066
NRS‐initial	−0.975	0.479	−0.192	−2.036	.063	0.813	1.230
MAO‐initial	0.951	0.102	0.859	9.359	.000**	0.859	1.164
*R* ^2^ (.906), adjusted *R* ^2^ (.877), *F* = 31.267, *p* = .000, Durbin–Watson (2.250)							

*Note*: An enter regression method was utilized.

Abbreviations: B, regression coefficient; MAO‐initial, initial maximum assisted opening; MPS, masseter muscle palpation score; NRS‐initial, initial numerically rated scale of pain; SE, standard error; TPS, temporalis muscle palpation score; VIF, variation inflation factor; β, standardized coefficient.

* *p* < .01

** *p* < .001.

The prognosis prediction Formula (2) for PFO‐final is the version without GCPS, sAA, and IL‐6.

In the multiple linear regression analysis, PFO‐final was positively correlated with MPS, PFO‐initial, and MAO‐initial and negatively correlated with TPS (*p* < .05) (Tables 5 and 6). A multiple linear regression analysis using NRS‐final as a dependent variable did not yield any significant results (*p* > .05, data not shown).

## 4 DISCUSSION

sAA is expressed higher in the MMP group than in the control group, and the higher the sAA value, the greater the restriction on daily life in the MMP (Figures 1 and 2). Several studies have reported on the correlation between symptoms of TMD or tension‐type headache (TTH) and sAA (Cozma et al., 2018; Kobayashi et al., 2017; McCarty et al., 1988). One study reported that sAA did not show significant differences between children with TMD and controls (Kobayashi et al., 2017). However, that study included both masticatory muscle and joint disorders, such as disc displacement. sAA is associated with a sympathetic activity that can directly affect muscles and has a higher correlation with MMP but not with all TMDs (Cozma et al., 2018; McCarty et al., 1988). TTH patients had significantly higher sAA levels, as well as symptoms associated with muscle pain (Vahedi et al., 2018). Furthermore, the sAA activity was correlated with the chronic indicator, GCPS, and disability days. These results suggest that patients with high sAA activity may experience more painful discomfort, implying the possibility of using sAA as an indicator of disability due to pain. This result agrees with other studies in which sAA is highly expressed in patients with chronic pain who have many limitations in their daily life due to pain (Arai et al., 2009; Shirasaki et al., 2007).

Regarding masseter and temporalis muscle pain, the IL‐6 level and response to NSAID treatment are different. Initially, high IL‐6 levels may require an active therapeutic approach using NSAIDs. The IL‐6 levels reflect the actual degree of inflammation in the muscles, and not the degree of pain caused by chronic or psychological conditions. In contrast, patients with initially low IL‐6 levels may have difficulty recovering because the patient's discomfort may be related to sympathetic hyperactivity or central sensitization of chronic pain and not the inflammation of the muscle itself. In addition, as shown in Figure 2, patients with low IL‐6 and high sAA have high discomfort in daily activity; therefore, a multidisciplinary approach may be appropriate. Patients with high IL‐6 and low sAA levels have less discomfort in life and respond well to NSAIDs. Table 4 shows the results of NSAID treatment in MPS and their correlation with IL‐6 levels. As shown in Tables 4, 5, 6, MPS (Tables 5 and 6) has a positive correlation with PFO, which is a treatment outcome evaluation factor, and it is pain related to acute factors (Table 4). Based on Tables 5 and 6, the MPS indicates a good prognosis. However, TPS showed a negative correlation with PFO (Tables 5 and 6), and it is chronic factors‐related pain (Table 4). Therefore, the therapeutic effect of NSAIDs may decrease, and the treatment prognosis is poor (Tables 5 and 6). In the case of TPS, a multifaced treatment modality can be recommended.

The contractility of the masseter muscle decreases faster than that of the temporalis muscle in repeated clenching tests (Hagberg & Hagberg, 1988). Thus, the masseter muscle becomes fatigued more easily than the temporalis muscle (Shirasaki et al., 2007). Larger amounts of MyHC‐I and MyHC‐IIA and smaller amounts of MyHC‐IIX in the temporalis muscle fibers make it slower and weaker but more resistant to fatigue than the masseter muscle (Isola et al., 2018; Korfage et al., 2005). Taken together, these findings indicate that the masseter muscle develops pain faster and is treated efficiently by NSAIDs, while the temporalis muscle develops pain after prolonged exposure to stimuli that exceed physiological tolerance (as in the chronic state). Therefore, patients with acute MMP tend to have masseter muscle pain only. If there is myalgia in the temporalis muscles, which are more resistant to fatigue, it may be considered that the patient suffers from chronic muscle pain due to overuse of the masticatory muscle for a long time.

IL‐6 positively correlates with age (Maggio et al., 2006), regardless of the presence of the disease. The number of IL‐6 receptors also increases with age (Sarkar & Fisher, 2006). In our study, the positive correlation between IL‐6 and age was not statistically significant (data not shown), possibly due to the small sample size. In the future, it will be possible to establish standard values of IL‐6 levels for each age that can be used for treatment selection by collecting data of various ages.

PSS showed no differences between the MMP and control groups, and there were no correlations with the other quantitative indicators measured, possibly because PSS relies on current psychological stress rather than physiological stress. Self‐reported perceived stress, such as PSS, may not always match the state of biological stress reactivity (Jung et al., 2015). In one study, PSS did not show any difference between patients with systemic lupus erythematosus and controls, but sAA showed differences between patients and controls (Peck et al., 2014). Our study also showed differences in sAA levels, but not in PSS. Therefore, sAA is suggested to be a more appropriate tool for measuring physiological stress in patients with MMP.

**Figure 4:**
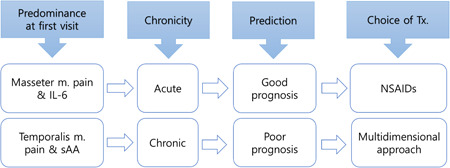
Prediction of prognosis. At the first visit, predominant masseter muscle pain and IL‐6 would mean acute pain from which we might predict a good prognosis with NSAIDs. Predominant temporalis muscle pain and sAA would mean chronic pain from which we might predict a poor prognosis that needs a multidimensional approach. IL‐6, interleukin‐6; NSAID, nonsteroidal anti‐inflammatory drug.

The main symptoms of MMP are limited mouth opening and pain (Okeson, 2019). Therefore, the improvement in these two symptoms, expressed as PFO and NRS, implies a good prognosis. PFO‐final correlates with several variables measurable during pretreatment. The wider the PFO‐final, the shorter the NSAID dosing period and the shorter the time required for treatment. These correlations are also consistent with the results presented in Table 5. In contrast, NRS‐final did not correlate with any variable other than NRS‐initial. Therefore, PFO‐final seems to be reliable for determining the comprehensive prognosis of MMP. Therefore, the prognostic prediction can be summarized as shown in Figure 4. The higher the masseter pain and IL‐6 levels at the first visit, the higher the meaning of acute pain. From that, we might predict a good prognosis with NSAIDs. Temporal pain and higher sAA levels indicate chronic pain. This allows the prediction of poor prognosis requiring a multidimensional approach.

From the prognosis prediction formulas above, quantitative data measured at pretreatment and PFO‐final can be predicted. In Formula (1), if the other conditions were constant, the PFO‐final increased by 1.531 mm when MPS increased by 1. Furthermore, the PFO‐final decreased by 2.339 mm when the TPS increased by 1. The prognosis prediction in Equation (2) is suitable for clinics that do not have systems for tests of GCPS, sAA, and IL‐6. If the other conditions were constant, the PFO‐final increased by 2.049 mm when MPS increased by 1. Furthermore, the PFO‐final decreased by 3.038 mm when the TPS increased by 1.

According to DC/TMD, MMP is mainly diagnosed based on the history of masticatory pain and masseter and temporalis muscle pain when palpated (Sarkar & Fisher, 2006). There are three subclasses of MMP: local myalgia, myofascial pain, and myofascial pain with referral. When myalgia is further subclassified as local myalgia, myofascial pain, or myofascial pain with referral, the latter diagnoses are based on using only the examination findings from palpation with the palpation pressure being held over the site for 5 s. According to the criteria of DC/TMD, the MMP in our study is local myalgia (Tanaka et al., 2014); however, we can subdivide the MMP in association with quantitative indicators and it was possible to suggest the effectiveness of treatment such as NSAIDs on MMP. This study is meaningful in that it provides helpful information when selecting a therapeutic agent for MMP.

### 4.1 Limitations of the study

This study demonstrates the association among quantitative indicators, clinical symptoms, and treatment results, which allows the classification of MMPs and provides effective treatment options for individuals. Fifteen subjects in the MMP group were lost to follow‐up due to failure to show up; therefore, consequent measurements of quantitative indicators could not be obtained. Therefore, studies should be conducted to develop various quantitative indicators with large sample sizes for optimum diagnosis and treatment. The structural limitation of this study was that IL‐6 was not analyzed in the control group. We could only obtain consent for stress assessment (e.g., PSS and sAA) and not for inflammation assessment (e.g., IL‐6) from most participants in the healthy control group. We plan to obtain consent from control groups and conduct extended studies to validate the prognosis prediction equation formula to establish specific personalized treatment guidelines. To analyze whether IL‐6, which can be expressed in diabetes and cardiovascular diseases, including various infections (Peck et al., 2014), is secreted according to the presence or absence of MMP, a healthy group was targeted. Furthermore, it is considered necessary to establish data considering changes in judgment and endurance of pain according to age. Therefore, further studies targeting sufficient subjects for each age group are needed in the future. Further studies with relaxed exclusion criteria and increased participant numbers are also needed to broaden the generalizability.

## 5 CONCLUSIONS

This study aimed to define a variety of quantitative indicators of MMP to provide efficient personalized treatment. As a result, we were able to reveal the diagnostic values of quantitative indicators, such as MPS and TPS. MPS and IL‐6 were associated with acute pain and can be used to predict a favorable prognosis. In addition, TPS, pain duration, and GCPS were associated with chronic pain, interference with daily life, and prediction of unfavorable prognosis. For instance, higher MPS and IL‐6 levels were indicated for NSAIDs as the first choice of treatment. When TPS, pain duration, and GCPS were high, a multidimensional approach was suggested with a longer treatment period. In addition, we developed Formulas (1) and (2) to predict the prognosis (final PFO) using several quantitative indicators at pretreatment. Notably, Formula (2) could be utilized using the indicators to avoid extra lab measurements (saliva or blood collection).

## AUTHOR CONTRIBUTIONS

*Conceptualization*: Sunhee Lee, Soo‐Min Ok, and Kyounga Cheon. *Methodology*: Sunhee Lee, Sung‐Hee Jeong, Yong‐Woo Ahn, and Hye‐Min Ju. *Data curation*: Sunhee Lee and Soo‐Min Ok. *Formal analysis*: Hye‐Min Ju and Byong‐Sop Song. *Software*: Hye‐Min Ju. *Investigation*: Sunhee Lee, Soo‐Min Ok, and Kyounga Cheon. *Writing – original draft*: Sunhee Lee and Soo‐Min Ok. *Writing – review and editing*: Donald Ho, Soo‐Min Ok, and Kyounga Cheon. *Funding acquisition*: Soo‐Min Ok and Kyounga Cheon.

## CONFLICT OF INTEREST

The authors declare no conflict of interest.

## Data Availability

The data supporting the findings of this study are available from the corresponding author upon reasonable request.
